# Reduced Expression of IFIH1 Is Protective for Type 1 Diabetes

**DOI:** 10.1371/journal.pone.0012646

**Published:** 2010-09-09

**Authors:** Kate Downes, Marcin Pekalski, Karen L. Angus, Matthew Hardy, Sarah Nutland, Deborah J. Smyth, Neil M. Walker, Chris Wallace, John A. Todd

**Affiliations:** Juvenile Diabetes Research Foundation/Wellcome Trust Diabetes and Inflammation Laboratory, Department of Medical Genetics, Cambridge Institute for Medical Research, University of Cambridge, Addenbrooke's Hospital, Cambridge, United Kingdom; La Jolla Institute of Allergy and Immunology, United States of America

## Abstract

IFIH1 (interferon induced with helicase C domain 1), also known as MDA5 (melanoma differentiation-associated protein 5), is one of a family of intracellular proteins known to recognise viral RNA and mediate the innate immune response. *IFIH1* is causal in type 1 diabetes based on the protective associations of four rare variants, where the derived alleles are predicted to reduce gene expression or function. Originally, however, T1D protection was mapped to the common *IFIH1* nsSNP, rs1990760 or Thr946Ala. This common amino acid substitution does not cause a loss of function and evidence suggests the protective allele, Ala^946^, may mark a haplotype with reduced expression of *IFIH1* in line with the protection conferred by the four rare loss of function alleles. We have performed allele specific expression analysis that supports this hypothesis: the T1D protective haplotype correlates with reduced *IFIH1* transcription in interferon-β stimulated peripheral blood mononuclear cells (overall *p* = 0.012). In addition, we have used multiflow cytometry analysis and quantitative PCR assays to prove reduced expression of IFIH1 in individuals heterozygous for three of the T1D-associated rare alleles: a premature stop codon, rs35744605 (Glu627X) and predicted splice variants, rs35337543 (IVS8+1) and rs35732034 (IVS14+1). We also show that the nsSNP, Ile923V, does not alter pre-mRNA levels of *IFIH1*. These results confirm and extend the new autoimmune disease pathway of reduced IFIH1 expression and protein function protecting from T1D.

## Introduction

Susceptibility to T1D was originally mapped to a common SNP, rs1990760 in the *IFIH1*, *FAP*, *GCA* and *KCNH7* region of chromosome 2q24 [1]. Further SNP typing revealed several variants across the region in high linkage disequilibrium (LD) (Figure S1) and statistically indistinguishable for T1D association. However, the nsSNP, rs1990760 or Thr946Ala, in exon 15 of *IFIH1*, was considered the most likely functional candidate due to the highly conserved amino acid substitution and the role of *IFIH1* in innate immunity, including mediating type 1 interferon production, which has been consistently reported to be associated with type 1 diabetes [1], [2], [3]. More recently, four rare or low frequency variants within *IFIH1* were associated with T1D, indicating the gene is causal [4] (Figure S2 and Table S1). The four variants share a common feature: the minor alleles, which have predicted functional implications, are protective for T1D, implying that reduced expression or function of IFIH1, which is known to be a receptor for viral RNA, protects against T1D. The common variant or haplotype is still associated with T1D after taking into account the rare variants, and vice versa (Table S1).

Experimental analysis of the common nsSNP, rs1990760/Thr946Ala, has revealed that the amino acid substitution does not alter the function of IFIH1, at least within the parameters and sensitivity of the assay employed [5], and evidence suggests rs1990760/Thr946Ala genotype correlates with mRNA levels [6]. In addition, it is the conserved allele that encodes Ala^946^, the presumed functional amino acid, not the derived allele, as is the case for the four low frequency variants (Table S1). Therefore, we hypothesise that Ala^946^ is not causal itself but a marker for common haplotypes carrying functional variants that reduce *IFIH1* transcription compared to other haplotypes with normal or higher expression. Two groups have previously attempted to correlate genotype at rs1990760/Thr946Ala with expression of *IFIH1* with only one identifying an association [6], [7]. These studies used quantitative PCR (qPCR) which has limited sensitivity for detecting small differences in transcript levels between different individuals with defined *IFIH1* genotypes. Therefore, we used allele-specific expression (ASE) analysis to detect transcriptional bias within individuals using genomic DNA from the same individual as a control. Most recently Zouk et al. [8] also applied an ASE approach to test the same hypothesis at *IFIH1* and found no evidence for allelic imbalance at rs1990760. Here, we have extended these analyses to induced *IFIH1* RNA using additional ASE assays and obtained positive results.

The two exonic T1D associated SNPs, Ile923Val and Glu627X, reduce the type I interferon response to Poly I:C, a viral dsRNA mimic, in complementation assays providing evidence of their function [5]. Here we have selected donors from a genotyped bioresource (www.cambridgebioresource.org.uk) that are heterozygous for one of the four T1D associated low frequency variants. Using qPCR and quantitative multicolor flow cytometric analysis, we have measured the protein expression, splicing and transcript levels to prove the functionality of the rare T1D variants in human immune cell subsets.

## Results

### Allele specific expression of the common *IFIH1* T1D haplotype

We used a sequencing based ASE assay [9] to correlate the pre-mRNA level of *IFIH1* with the common haplotypes marked by the T1D associated nsSNP Thr946Ala. Subjects selected for ASE analysis had no low frequency *IFIH1* T1D alleles and were heterozygous at both common *IFIH1* nsSNPs, Thr946Ala (rs1990760 A/G) and A843H (rs3747517 C/T). Phasing genotypes of 9,970 control samples (using SNPHAP, http://www-gene.cimr.cam.ac.uk/clayton/) revealed the T1D protective minor alleles of rs1990760 and rs3747517 always reside on the same haplotype in individuals heterozygous at both SNPs. Genomic and cDNA from each donor was amplified, sequenced and peak heights analysed and normalised using the surrounding peaks [9]. Owing to concerns over biases caused by RNA secondary structure [10], cDNA was synthesised using PBMC purified pre-mRNA, poly A selected by priming with oligo-dT and using total RNA fragmented and primed using random hexamers.

In nine samples no allelic specific bias for the common *IFIH1* T1D associated haplotype was observed within the PBMC pre mRNA of the poly A selected samples (*p* = 0.12) or the fragmented cDNAs (*p* = 0.60) using the two SNP assays for rs1990760 and rs3747517 (Figure S3).

Stimulation of PBMCs with IFN-β induces the transcription of *IFIH1* [11] and a positive feedback loop, through IFN regulatory factor 7, induces *IFN-β* expression [12], [13]. On stimulation with 1,000 U/ml IFN-β we measured a 27 fold and 40 fold induction of *IFIH1* and IFN-β transcription respectively (Figure S4). We hypothesised that the regulatory loop not only increases IFIH1 expression, but may also amplify any transcriptional bias, if present.

Assays for rs1990760 and rs3747517 were used to measure the allelic bias in eight oligo-dT primed pre mRNA samples from PBMCs stimulated with IFN-β for 4 hours. To provide additional ASE data, an assay was designed for a third *IFIH1* SNP, rs13023380. In all ASE individuals the minor allele of rs13023380 (G) was on the T1D protective haplotype, also marked by the minor alleles of rs1990760 (G) and rs3747517 (T).

**Figure 1:**
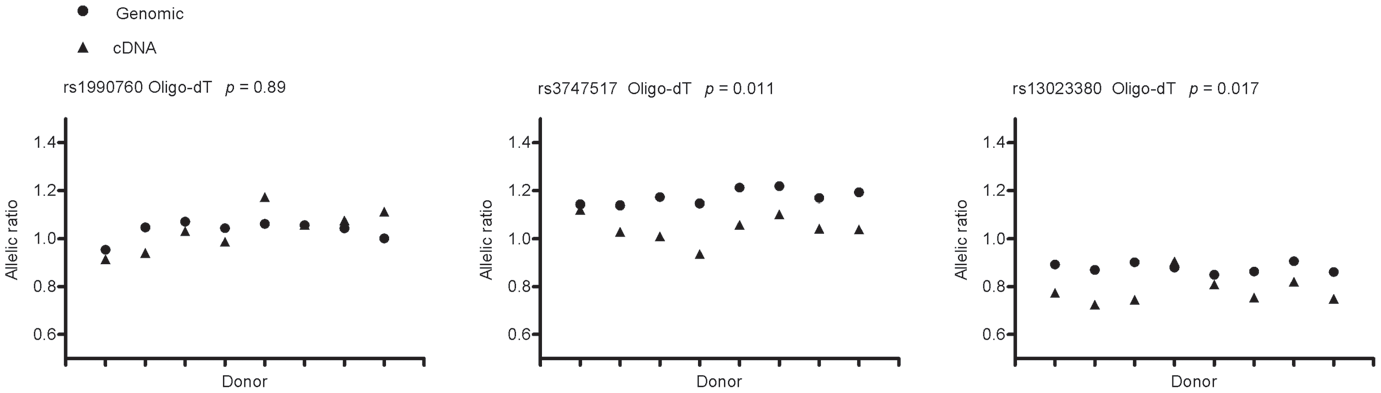
Allele specific expression of *IFIH1* pre mRNA transcript for the common T1D haplotype in IFN-β stimulated PBMCs. The mean allelic ratio is a measure of the ratio of the sequencing trace peak heights for the heterozygous SNP within genomic and cDNA from each individual (overall sign-rank *p* = 0.012).

ASE was observed in *IFIH1* poly A selected pre mRNA from INF-β stimulated PBMCs using the rs3747517 and rs13023380 SNP assays (Figure 1). The T1D associated haplotype correlated with reduced transcription of *IFIH1* (overall sign-ranked test *p* = 0.012). Fragmenting RNA prior to cDNA synthesis does not alter the ASE observed at rs1990760 (*p* = 0.48)(Figure S5).

### The rare *IFIH1* SNPs associated with T1D are functional

**Figure 2:**
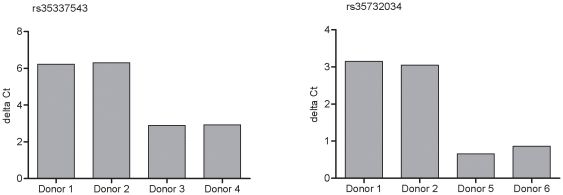
Detection of aberrant splicing of *IFIH1* mRNA for the two rare SNPs predicted to alter splicing. Levels of *IFIH1* pre mRNA and spliced RNA transcript were measured using qPCR assays designed to the corresponding exon to intron and exon to exon sequence. The delta Ct is calculated using the pre mRNA transcript Ct minus the mRNA transcript Ct. A larger delta Ct indicates more pre-mRNA and less mRNA transcript of *IFIH1*. Donor 1 and 2 are control individuals, donor 3 and 4 heterozygous for rs35337543 (IVS8+1) and donor 5 and 6 are heterozygous for rs35732034 (IVS14+1).

The two T1D-associated intronic SNPs within *IFIH1*, rs35337543 (IVS8+1) and rs35732034 (IVS14+1), alter the 5′ base of intron 8 and intron 14 consensus splice sequences, respectively, and were predicted to alter splicing of the intron [4]. We recalled six healthy donors, from the Cambridge BioResource, two individuals with no known rare *IFIH1* variants, two individuals heterozygous for rs35337543 (IVS8+1) and two individuals heterozygous for rs35732034 (IVS14+1). qPCR assays were designed to measure the level of spliced and unspliced transcript, by designing primer pairs and probes specific to the *IFIH1* pre-mRNA and mRNA at the appropriate splicing junction. The qPCR data indicated that PBMCs from individuals heterozygous for the splice SNPs had more pre-mRNA transcript and less mRNA transcript compared to the control donor at these splicing junctions (Figure 2). These data confirm that the two SNPs, rs35337543 (IVS8+1) and rs35732034 (IVS14+1), result in aberrant splicing of the *IFIH1* transcript.

The T1D-associated *IFIH1* SNP, rs35744605 (Glu627X), introduces a premature stop codon into the transcript. Using the mRNA qPCR assays described above we measured the levels of transcript within individuals heterozygous for the SNP, rs35744605 (Glu627X). The premature stop codon did not alter the level of *IFIH1* mRNA detected within resting human PBMC (Table S2). This is perhaps not unexpected since nonsense mediated decay (NMD) of transcripts containing premature stop codons is initiated during the pioneering round of translation within the cytoplasm. Resting immune cells do not express high levels of *IFIH1* (Figure S7) suggesting the low turnover of translation results in small differences in mRNA levels between individuals with Stop^627^ and Glu^627^, and these are undetectable by qPCR.

**Figure 3:**
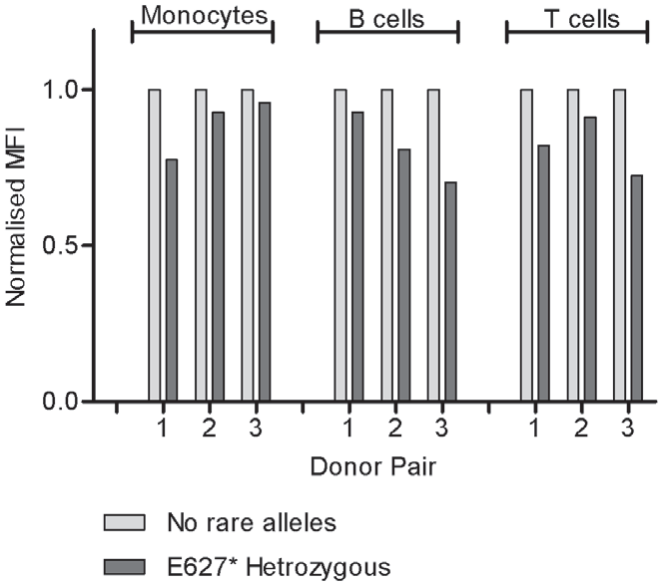
Reduced IFIH1 expression within monocytes, B and T cells in donors heterozygous for the rare premature stop codon SNP, rs35744605 (Glu627X). Mean fluorescent intensity (MFI) of IFIH1 expression for each donor heterozygous for rs35744605 is normalised to the MFI of the paired control individual.

To detect the reduced expression of *IFIH1* due to the premature stop codon we measured the level of the IFIH1 protein within cells. We developed an intracellular staining protocol to detect IFIH1 using multicolour flow cytometry to quantify the amount of IFIH1 within subsets of peripheral immune cells. IFIH1 protein is detected in resting monocytes, B and T cells and upon stimulation with IFN-β (Figure S6 and S7). To demonstrate that the SNP, rs35744605 (Glu627X), causes reduced expression of IFIH1, three pairs of donors were assayed. Within each pair, one individual did not carry any known *IFIH1* variants and the other was heterozygous for the stop codon SNP. IFIH1 expression was reduced in cell subsets in individuals heterozygous for the stop codon (Figure 3). The reduced expression observed in samples heterozygous for rs35744605 (Glu627X) is amplified with IFN-β stimulation within cell subsets (Figure S8) and in total protein lysates of PBMCs (Figure S9).

**Figure 4:**
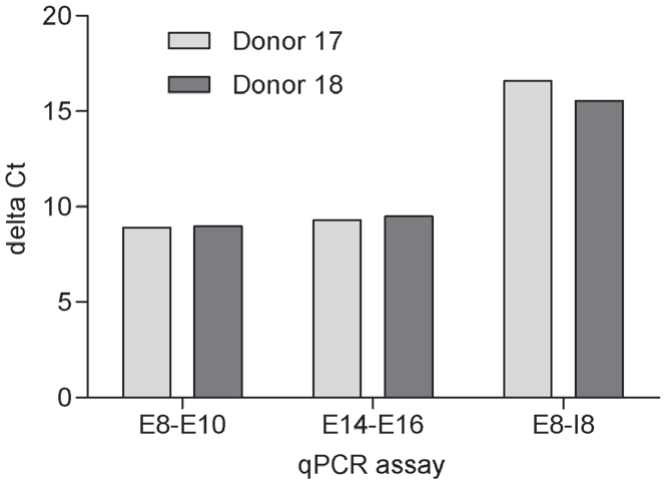
Levels of pre-mRNA and mRNA transcript of *IFIH1* are not altered by the rare nsSNP, rs35667974, Ile923Val. Delta Ct calculated using the *IFIH1* qPCR minus the single copy gene β2 microglobulin (B2M) qPCR. Donor 17 is the control individual and donor 18 is heterozygous for Ile923Val. E8-E10: exon 8 to exon 10 qPCR assay, E14-E16: exon 14 to exon 16 qPCR assay and E8-I8: exon 8 to intron 8 qPCR assay.

Cells expressing constructs containing the rare nsSNP allele Val^923^ at rs35667974 (Ile923Val) have a reduced type I IFN response to Poly I:C [5], indicating that this allotype reduces function of IFIH1. To investigate if this T1D associated rare nsSNP also alters expression or splicing of *IFIH1* RNA within human immune cells we used the qPCR assays described above to measure pre-mRNA and mRNA levels in cDNA synthesised using RNA purified from PBMCs of an individual selected to be heterozygous for Ile923Val. Pre mRNA or mRNA levels did not correlate with Ile923Val (Figure 4) suggesting that, within this individual, Ile923Val did not alter expression of *IFIH1* in a major way. ASE analysis of pre-mRNA at Ile923Val did not show any allelic imbalance (data not shown). Since Val^923^ does not alter dsRNA binding [5], we predict that the amino acid substitution reduces the efficiency of IFIH1 interactions, possibly with interferon-beta promoter stimulator protein-1 (MAVS, mitochondrial antiviral signaling protein, also known as *IPS-1*, VISA and Cardif) during anti-viral signaling [14].

## Discussion

IFIH1 (interferon induced with helicase C domain 1), also known as MDA5 (melanoma differentiation-associated protein 5), is one of a family of intracellular proteins known to recognise viral RNA and mediate an immune response [15]. The *IFIH1* receptor is specific for the double stranded intermediate generated during replication of single stranded, positive sense picornaviruses [16] and negative sense Paramyxoviridae [17]. This, and the established seasonality of T1D diagnosis, increasing incidence of T1D [18] and reports that Coxsackie infection is associated with T1D [19], suggest that several viruses may be involved in the aetiology of the disease [3].

The verification of the predicted loss of function of the four rare SNPs (here and Shigemoto T., et al. [5]) confirms that reduced IFIH1 protein and function protects against T1D. We also hypothesised that the protective allele of the common T1D nsSNP, rs1990760, may mark haplotypes with reduced expression of IFIH1, in line with the loss of function conferred by the protective alleles of the low frequency T1D variants.

qPCR has limited sensitivity for detecting small differences in transcript levels between individuals, and therefore, we used an ASE method to measure allelic imbalance within individuals heterozygous for SNPs that mark the T1D haplotype. Here we present evidence that the protective T1D associated haplotype correlates with reduced transcription of *IFIH1* that is reproducibility observed upon induction of *IFIH1* transcription using IFN-β (Figure 1).

No allelic bias was detected in resting PBMCs (Figure S3) possibly due to the low levels of transcript available for amplification or that the effect of allelic variation on *IFIH1* transcription only manifests in conditions of stimulation, stress or infection in vivo. Biological and/or experimental factors could account for the lack of bias observed at the SNP rs1990760. Interestingly, rs1990760 lies within exon 14 at the 3′ of the gene while the SNPs rs3747517 and rs13023380 are towards the 5′ end of the gene. This observation may account for the discordance between two of the previous attempts to correlate Ala^946^ of rs1990760 with IFIH1 gene expression by qPCR of un-fragmented RNA hexamer primed cDNA [6], [20]. One group did not observe an expression difference using a quantitative PCR assay at the 3′ end of the gene [20], while the other study measured mRNA at the 5′ end of the gene and showed lower *IFIH1* expression for haplotypes bearing Ala^946^, a result consistent with ours [6]. We did not observe allelic imbalance at rs1990760 using an ASE approach in steady state or induced RNA, consistent with the recent null result from Zouk et al. [8]. These results highlight the importance of analysing multiple SNPs in a gene for ASE studies [21].

Further functional studies will help elucidate the mechanisms by which normal or wild type function of IFIH1 predisposes to pancreatic autoimmunity. IFIH1-mediated type 1 interferon responses within monocytes, T and B cells could maintain the activation, expansion or differentiation of autoreactive T cells [4]. In addition, normal or activated anti viral responses in T1D susceptible individuals could be permissive for apoptosis of infected pancreatic β cells, which express high levels of *IFIH1* RNA, and induce type 1 interferon signaling, increasing HLA class I expression on β cells, thereby increasing CD8 T cell mediated destruction [2], [3].

## Materials and Methods

### Donors and genotypes

Blood was collected from donors selected from a pre-genotyped bioresource and processed within 4 hours (www.cambroidgebioresource.rg.uk). Within each experiment samples were processed in parallel using the same reagents. ASE donors were heterozygous at rs1990760 and rs3747517 and heterozygous for rs13023380 in the IFN-β stimulated ASE samples. Genotypes for all other donors at the five *IFIH1* SNPs are listed in Table S3.

### Study ethics

Subjects were recruited from the Cambridge BioResource (www.cambridgebioresource.org.uk) as part of the ‘Genes and Mechanisms of Type 1 Diabetes’ study. The study has ethical approval from the NHS Cambridgeshire Research Ethics Committee. All data and samples are treated as confidential. Blood and saliva samples are stored identified by a unique bar code only and volunteers are free to withdraw from the project at any time.

### PBMC isolation and preparation of DNA and RNA

PBMCs were purified from heparinised blood diluted 1∶1 in phosphate buffered saline (PBS) (without Ca^2+^ and Mg^2+^, GIBCO, Invitrogen). Fifteen ml aliquots of the diluted blood were layered onto 10 ml aliquots of Lympholyte® (Cedarlane Laboratories Ltd) and were centrifuged at 800× g for 20 min at room temperature with no brake. The PBMC layer was washed twice with PBS, with centrifugation at 300× g for 10 min at 4°C.

Total RNA from PBMCs was extracted using TRIzol® Reagent (Invitrogen) and DNaseI-treated using the RNeasy Mini kit (QIAGEN), according to the manufacturers' instructions. RNA quality was assessed using a Bioanalyzer (Agilent technologies) and quantitated using a NanoDrop spectrophotometer (Thermo Scientific). Prior to cDNA synthesis the RNA was either left unfragmented or fragmented using a chemical fragmentation reagent according to the manufacturers protocol (Ambion). First strand complementary DNA synthesis was carried out on 1 µg of RNA using Superscript™ III RT kit and using either oligo-dT for the un-fragmented or random hexamers (Invitrogen) for the fragmented RNA samples. The 150–350 base-pair fragments of cDNA primed using hexamer were selected using gel purification. Genomic DNA was extracted from an aliquot of blood by chloroform extraction.

### Allele specific expression assays (ASE)

Primers flanking rs35667974, rs1990760 and rs13023380 were used to amplify cDNA and genomic DNA by PCR using AmpliTaq Gold™ (ABI) in duplicate. For sequencing based ASE, duplicate PCR products were mixed and gel purified, sequenced using BigDye Terminator chemistry (Applied Biosystems) and resolved using an Applied Biosystems 3730XL DNA analyzer. Up to four sequence traces per PCR product were analysed using PeakPicker software as described by Bing Ge [9] and used to estimate the allelic ratio. The geometric mean of the replicate allelic ratios was used in the Wilcoxon matched-pairs signed-rank test [22] to ask if the distribution differed between the matched genomic and cDNA sample pairs. The allelic ratios are correlated within samples across SNPs, therefore the geometric means of allelic ratios could be calculated across SNPs to perform an overall test of whether the allelic ratio distribution of cDNA and genomic differed, again using a Wilcoxon signed rank test.

### Quantitative PCR assays (qPCR)

qPCR primers and probe were designed to the pre mRNA and mRNA sequences flanking the SNPs rs35337543 and rs35732034 (Table S4). Probes were labeled with the fluorochrome FAM and were quenched using TAMRA. qPCR assays contained 5 µl of 1∶10 dilution of oligo-dT primed cDNA, prepared as described above, in 20 µl assays. Ct values were measured using a 7900HT ABI prism (ABI) and analysis was performed using SDS v2.1 software (ABI). qPCR reactions were run in duplicate and delta Cts for the splice assays were calculated using the Ct of the pre mRNA qPCR minus the Ct of the mRNA qPCR. Delta Ct for the transcript level qPCRs were calculated using the *IFIH1* qPCR Ct value minus the single copy gene β2 microglobulin (B2M) qPCR Ct value.

### Flow cytometry

PBMCs were purified as described above. For IFN-β stimulation, 1,000 U/ml of IFN-β (R&D Biosystems) was used per two million PBMCs in X-VIVO 15 medium (Lonza Walkersville, Inc.) supplemented with heat inactivated human AB serum (Sigma) at 37°C for 6 h or overnight.

PBMCs were stained for 30 min on ice using allophycocyanin (APC)-conjugated anti-CD16, Alexa Fluor 700-conjugated anti-CD3, PE-Cy7 conjugated anti-HLA-DR, PE-conjugated CD19 (BioLegend) and Pacific Blue-conjugated anti-CD14 (BD Biosciences) according to maunufacturer's instructions. Cells were fixed at 37°C for 10 min using BD Phosphflow Fix Buffer 1 (BD Biosciences) then permeabilised with ice cold BD Phosphflow Perm Buffer III (BD Biosciences) for 30 min on ice. Cells were washed with BD Pharmingen Stain Buffer (BD Biosciences) for 5 min at 500× g, with an extra wash following permeabilisation and intracellular staining. Cells were blocked and incubated with a primary anti-*IFIH1* rabbit polyclonal (Prestige) for 60 min on ice and washed twice before addition of Alexa Fluor 488-conjugated goat anti-rabbit IgG (Invitrogen) at 1∶200 dilution for 30 min on ice. Cells were analysed using a BD LSRII Flow Cytometer with BD FACSDiVa Software (BD Biosciences) and mean fluorescence intensity (MFI) data was obtained via FlowJo (Tree Star, Inc.).

## Supporting Information

Table S1 Association analysis of each of the *IFIH1* SNPs contribution to T1D association in the region. Logistic regression analysis was performed using a complete data set of 7,024 cases and 8,844 control samples. The 1df test result was obtained by adding each SNP to a logistic regression model containing the data for the other four SNPs. MAF  =  Minor Allele Frequency; OR  =  Odds Ratio; 95% CI  =  95% Confidence Interval; df  =  degree of freedom. Adapted from Nejentsev S, Walker N, Riches D, Egholm M, Todd JA, (2009). Rare variants of *IFIH1*, a gene implicated in antiviral responses, protect against type 1 diabetes. Science 324: 387-389.
(0.04 MB DOC)

Table S2 Expression of *IFIH1* mRNA is not altered by the SNP, rs35744605 (Glu627X), that encodes for a premature stop codon. Donor 7 and 8 are control individuals and donor 9 and 10 are heterozygous for rs35744605 (Glu627X). Delta Ct is calculated using the *IFIH1* qPCR minus the single copy gene β2 microglobulin (B2M) qPCR.
(0.03 MB DOC)

Table S3 Genotypes of donors used in functional assays.
(0.05 MB DOC)

Table S4 Primer and probe sequences.
(0.03 MB DOC)

Figure S1 Recombination rate in the IFIH1 gene region on chromosome 2. Genome-wide recombination rate from Phase 2 HapMap, estimated from phased haplotypes in HapMap Release 22 (NCBI 36), remapped to GRCh37 and presented in T1DBase. Hulbert EM, et al. (2007). T1DBase: integration and presentation of complex data for type 1 diabetes research. Nucleic Acids Res 35: D742-746.
(0.10 MB TIF)

Figure S2 Schematic presentation of the IFIH1 protein (green), its domains (yellow) and T1D variants. The four rare variants, rs35667974/Ile923Val, rs35337543/IVS8+1, rs35744605/Glu627X,and rs35732034/IVS14+1 and the common variant, rs1990760/Thr946Ala, that are independently associated with T1D and their positions are shown. CARD - caspase recruitment domain. Adapted from Nejentsev S, Walker N, Riches D, Egholm M, Todd JA, (2009). Rare variants of *IFIH1*, a gene implicated in antiviral responses, protect against type 1 diabetes. Science 324: 387-389.
(0.12 MB TIF)

Figure S3 No allele specific bias was observed in *IFIH1* pre mRNA transcript using poly A selected or fragmented cDNA with rs1990760 or rs3747517 ASE assays. Sign-rank test oligo-dT samples versus genomic *P* = 0.12 and fragmented cDNAs versus genomic *P* = 0.60.
(0.10 MB TIF)

Figure S4 Pre mRNA levels of *IFIH1* and *IFNB1*, the gene encoding IFN-β, increase and plateau in PBMCs stimulated with 1000 U/ml of IFN-β for 6 hours. Ct values for the gene specific assays are normalised using a qPCR assay for β2 microglobulin.
(0.10 MB TIF)

Figure S5 No allele specific bias is observed using the rs1990760 ASE assay in cDNA of fragmented RNA primed with hexamer from PBMCs stimulated with IFN-β (*P* = 0.64).
(0.10 MB TIF)

Figure S6 Mean fluorescence intensity (MFI) of IFIH1 protein expression in resting monocytes, B and T cells of the three paired control individuals.
(0.10 MB TIF)

Figure S7 Expression of IFIH1 protein increases with IFN-β stimulation at 6 hours and overnight in monocytes, B and T cells of one paired control individual.
(0.10 MB TIF)

Figure S8 The reduced expression of IFIH1 protein due to the stop codon SNP, rs35744605 (Glu627X), is amplified with IFN-β stimulation within the cell subsets. Normalised MFI of IFIH1 expression within monocytes, B and T cells stimulated with 1,000 U/ml IFN-β overnight. The MFI of individuals heterozygous for the stop codon SNP is normalised to the MFI of the paired control individual.
(0.11 MB TIF)

Figure S9 Reduced expression of IFIH1 due to the stop codon SNP, rs35744605 (Glu627X), may be observed by western blot of protein lysates from resting PBMCs and PBMCs stimulated with IFN-β for 6 hours and overnight.
(0.10 MB TIF)
