# Supplementary material for: Reduced Expression of IFIH1 Is Protective for Type 1 Diabetes
Source: PLoS One. 2010 Sep 9;5(9):e12646. doi: 10.1371/journal.pone.0012646 (PMC2936573; doi:10.1371/journal.pone.0012646)
Supplement: Table S1 — Association analysis of each of the IFIH1 SNPs contribution to T1D association in the region. Logistic regression analysis was performed using a complete data set of 7,024 cases and 8,844 control samples. The 1df test result was obtained by adding each SNP to a logistic regression model containing the data for the other four SNPs. MAF = Minor Allele Frequency; OR = Odds Ratio; 95% CI = 95% Confidence Interval; df = degree of freedom. Adapted from Nejentsev S, Walker N, Riches D, Egholm M, Todd JA, (2009). Rare variants of IFIH1, a gene implicated in antiviral responses, protect against type 1 diabetes. Science 324: 387-389. (0.04 MB DOC) [file pone.0012646.s001.doc]

**Table S1.**

| SNP |  | Conserved allele | Minor allele | %MAF in controls | OR  (95%CI) | 1df P-value |
| --- | --- | --- | --- | --- | --- | --- |
| rs1990760 | Thr946Ala | G | G | 39.29 | 0.85  (0.81-0.89) | 2.6 x 10^-11^ |
| rs35667974 | Ile923Val | A | G | 2.13 | 0.57  (0.47-0.69) | 1.4 x 10^-8^ |
| rs35337543 | IVS8+1 | G | C | 1.45 | 0.66  (0.53-0.81) | 1.1 x 10^-4^ |
| rs35744605 | Glu627X | G | T | 0.95 | 0.56  (0.42-0.79) | 5.2 x 10^-4^ |
| rs35732034 | IVS14+1 | G | A | 0.70 | 0.71  (0.56-0.91) | 6.9 x 10^-3^ |
