# Supplementary material for: Reduced Expression of IFIH1 Is Protective for Type 1 Diabetes
Source: PLoS One. 2010 Sep 9;5(9):e12646. doi: 10.1371/journal.pone.0012646 (PMC2936573; doi:10.1371/journal.pone.0012646)
Supplement: Table S2 — Expression of IFIH1 mRNA is not altered by the SNP, rs35744605 (Glu627X), that encodes for a premature stop codon. Donor 7 and 8 are control individuals and donor 9 and 10 are heterozygous for rs35744605 (Glu627X). Delta Ct is calculated using the IFIH1 qPCR minus the single copy gene β2 microglobulin (B2M) qPCR. (0.03 MB DOC) [file pone.0012646.s002.doc]

# Table S2.

|  | Exon 8 to Exon 10  delta Ct |
| --- | --- |
| Donor 7 | 8.68 |
| Donor 8 | 9.39 |
| Donor 9 | 8.74 |
| Donor 10 | 9.38 |
|  |  |
|  | Exon 14 to Exon 16  delta Ct |
| Donor 7 | 9.00 |
| Donor 8 | 9.58 |
| Donor 9 | 9.95 |
| Donor 10 | 9.78 |
