# Supplementary material for: Reduced Expression of IFIH1 Is Protective for Type 1 Diabetes
Source: PLoS One. 2010 Sep 9;5(9):e12646. doi: 10.1371/journal.pone.0012646 (PMC2936573; doi:10.1371/journal.pone.0012646)
Supplement: Table S3 — Genotypes of donors used in functional assays. (0.05 MB DOC) [file pone.0012646.s003.doc]

**Table S3.**

| Donor | Experiment | rs1990760 | rs35337543 | rs35732034 | rs35744605 | rs35667974 |
| --- | --- | --- | --- | --- | --- | --- |
|  |  | Thr946Ala | intron8,+1splice | intron14,+1splice | Glu627X | Ile923Val |
| 1 | Splice qPCR | AA | GG | GG | GG | AA |
| 2 | Splice qPCR | AA | GG | GG | GG | AA |
| 3 | Splice qPCR | AA | **GC** | GG | GG | AA |
| 4 | Splice qPCR | AA | **GC** | GG | GG | AA |
| 5 | Splice qPCR | AA | GG | **GA** | GG | AA |
| 6 | Splice qPCR | AA | GG | **GA** | GG | AA |
| 7 | Stop qPCR | AA | GG | GG | GG | AA |
| 8 | Stop qPCR | AA | GG | GG | GG | AA |
| 9 | Stop qPCR | AA | GG | GG | **GA** | AA |
| 10 | Stop qPCR | AA | GG | GG | **GA** | AA |
| 11 | Stop Flow | AA | GG | GG | GG | AA |
| 12 | Stop Flow | AA | GG | GG | **GA** | AA |
| 13 | Stop Flow | AA | GG | GG | GG | AA |
| 14 | Stop Flow | AA | GG | GG | **GA** | AA |
| 15 | Stop Flow | AA | GG | GG | GG | AA |
| 16 | Stop Flow | AA | GG | GG | **GA** | AA |
| 17 | nsSNP qPCR | AG | GG | GG | GG | AA |
| 18 | nsSNP qPCR | AG | GG | GG | GG | **AG** |
