# Supplementary material for: Reduced Expression of IFIH1 Is Protective for Type 1 Diabetes
Source: PLoS One. 2010 Sep 9;5(9):e12646. doi: 10.1371/journal.pone.0012646 (PMC2936573; doi:10.1371/journal.pone.0012646)
Supplement: Table S4 — Primer and probe sequences. (0.03 MB DOC) [file pone.0012646.s004.doc]

**Table S4.**

| rs1990760_ASE.F | TCCAATAACCCATGAACTACACA |
| --- | --- |
| rs1990760_ASE.R | TGCAATTAAACATTCCTGTTGG |
| rs3747517_ASE.F | AGCTGATGAGAGCACCTACGTC |
| rs3747517_ASE.R | CGCCATTTTTAAATGAAAATCAA |
| rs13023380_ASE.F | TTCCTCTAGGGAAAATATCAAAGC |
| rs13023380_ASE.R | TAAAAAGGCCTTCAACCTCTTG |
| rs35732034_E.F | TGACCCCAGAATTCAAGGAA |
| rs35732034_E.R | AAGGCAAATCTAAGCCTTTGTG |
| rs35732034_E.P | GCCAGGCTTGGGGAACAATG |
| rs35732034_I.F | TGCAGTGTGCTAGCCTGTTC |
| rs35732034_I.R | CTTCACCCCTTGTGGAAAAA |
| rs35732034_I.P | TGCATCACGTCAATATGACCCCA |
| rs35337543_E.F | GATGCAACCAGAGAAGATCCAT |
| rs35337543_E.R | ACACGTTCTTTGCGATTTCC |
| rs35337543_E.P | CAACCCTATGAACAATGGGCCA |
| rs35337543_I.F | GTGCCAATCTTGATGCATTT |
| rs35337543_I.R | GCCTTTGCCATCTTTCTACTG |
| rs35337543_I.P | TGCCATTGCAGATGCAACCA |
| IFN-beta.F | AAACTCATGAGCAGTCTGCA |
| IFN-beta.R | AGGAGATCTTCAGTTTCGGAGG |
| IFN-beta.P | AGTACAGTCACTGTGCCTGGACCAT |
