# Supplementary figures and images for: Reduced Expression of IFIH1 Is Protective for Type 1 Diabetes
Source: PLoS One. 2010 Sep 9;5(9):e12646. doi: 10.1371/journal.pone.0012646 (PMC2936573; doi:10.1371/journal.pone.0012646)

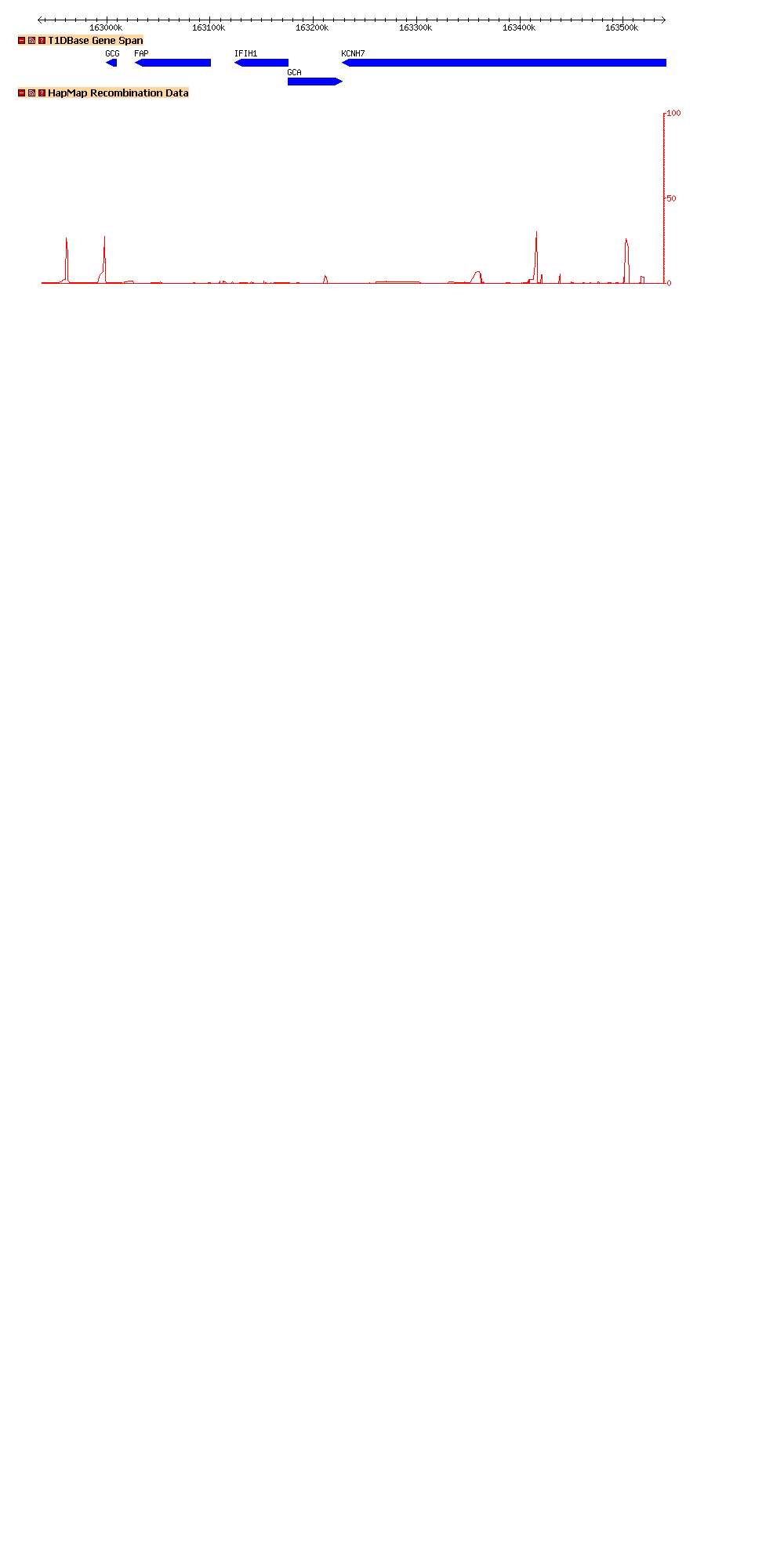

Supplement: Figure S1 — Recombination rate in the IFIH1 gene region on chromosome 2. Genome-wide recombination rate from Phase 2 HapMap, estimated from phased haplotypes in HapMap Release 22 (NCBI 36), remapped to GRCh37 and presented in T1DBase. Hulbert EM, et al. (2007). T1DBase: integration and presentation of complex data for type 1 diabetes research. Nucleic Acids Res 35: D742-746. (0.10 MB TIF) [file pone.0012646.s005.tif]

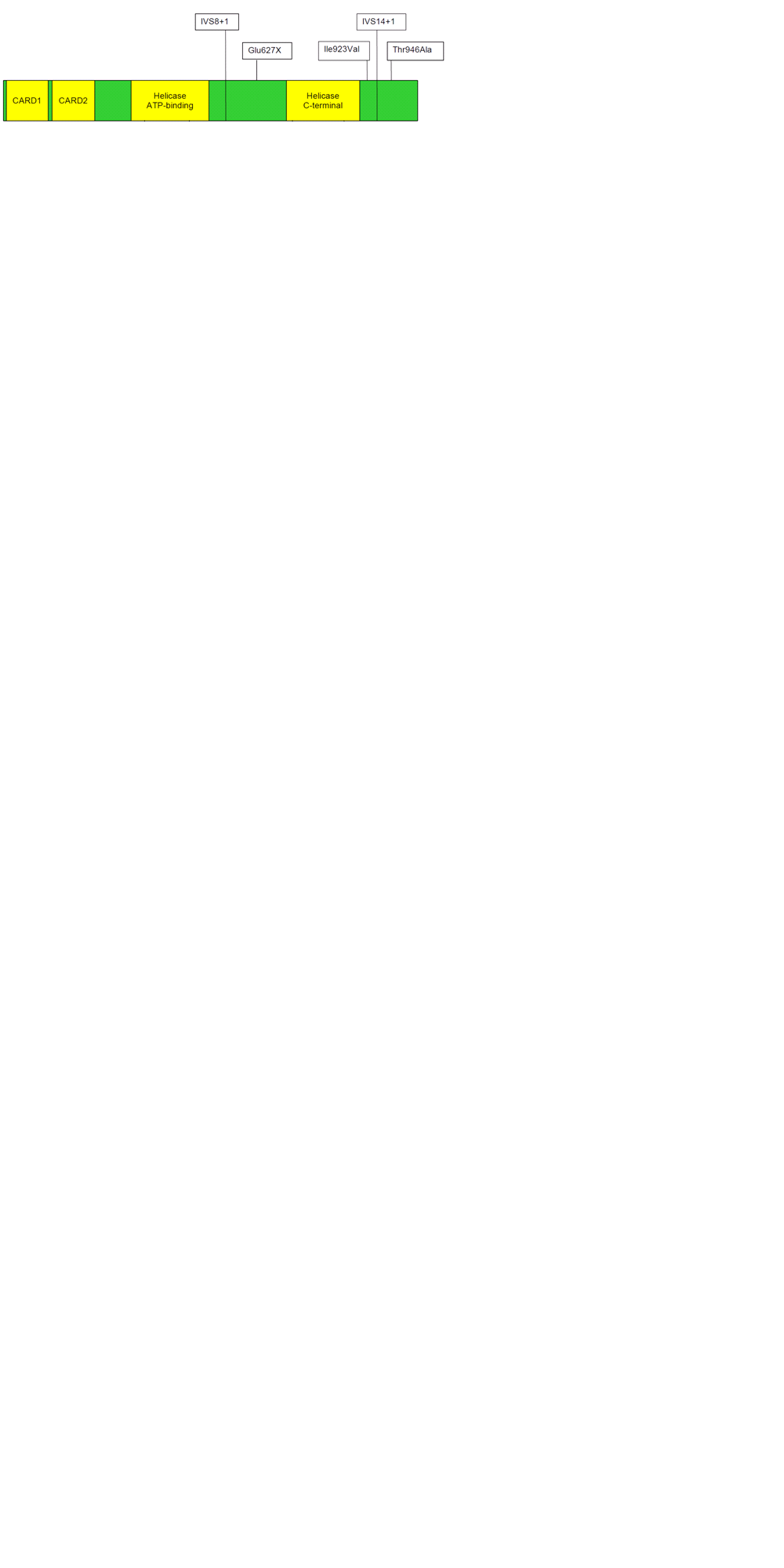

Supplement: Figure S2 — Schematic presentation of the IFIH1 protein (green), its domains (yellow) and T1D variants. The four rare variants, rs35667974/Ile923Val, rs35337543/IVS8+1, rs35744605/Glu627X,and rs35732034/IVS14+1 and the common variant, rs1990760/Thr946Ala, that are independently associated with T1D and their positions are shown. CARD - caspase recruitment domain. Adapted from Nejentsev S, Walker N, Riches D, Egholm M, Todd JA, (2009). Rare variants of IFIH1, a gene implicated in antiviral responses, protect against type 1 diabetes. Science 324: 387-389. (0.12 MB TIF) [file pone.0012646.s006.tif]

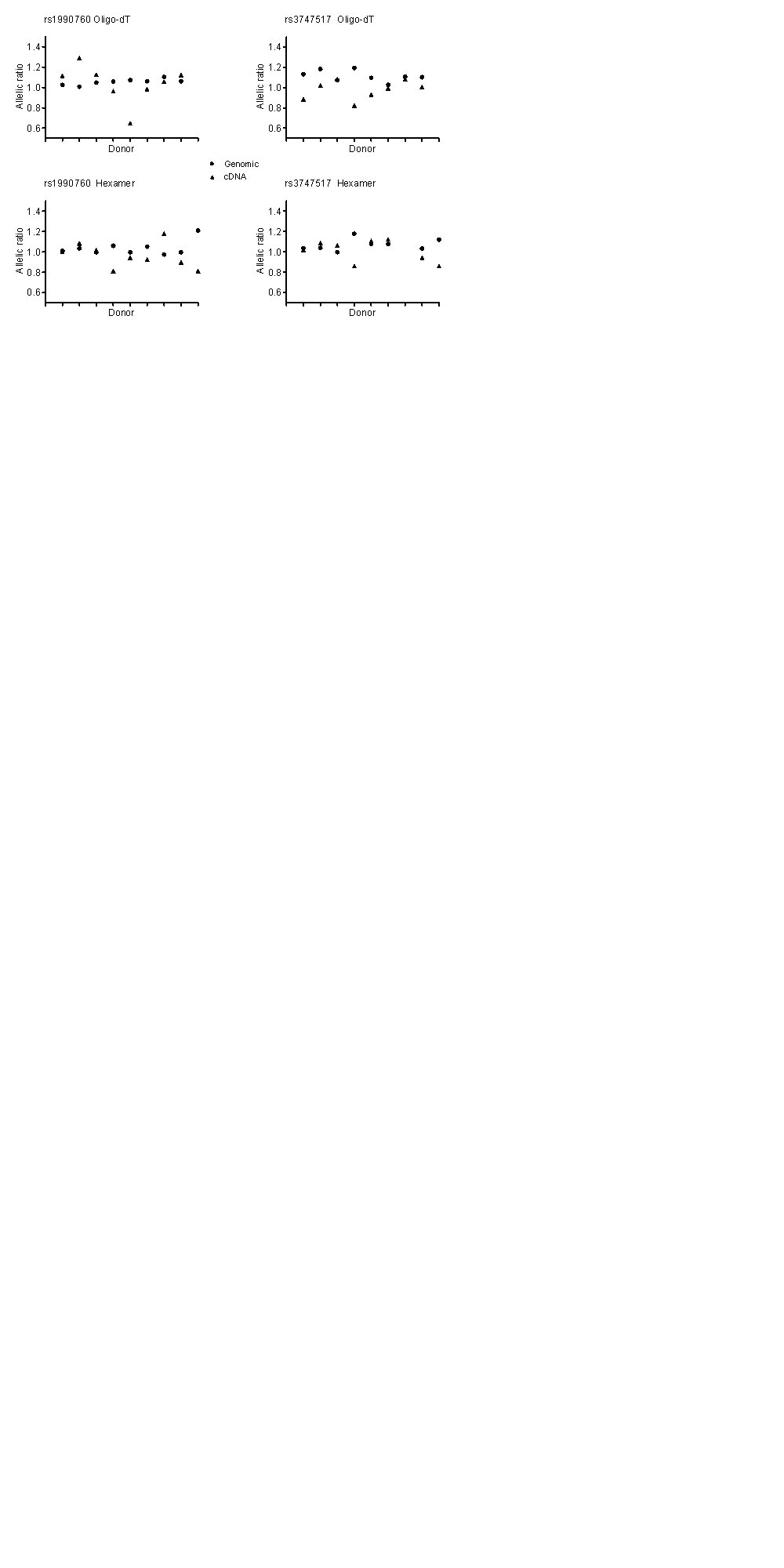

Supplement: Figure S3 — No allele specific bias was observed in IFIH1 pre mRNA transcript using poly A selected or fragmented cDNA with rs1990760 or rs3747517 ASE assays. Sign-rank test oligo-dT samples versus genomic P = 0.12 and fragmented cDNAs versus genomic P = 0.60. (0.10 MB TIF) [file pone.0012646.s007.tif]

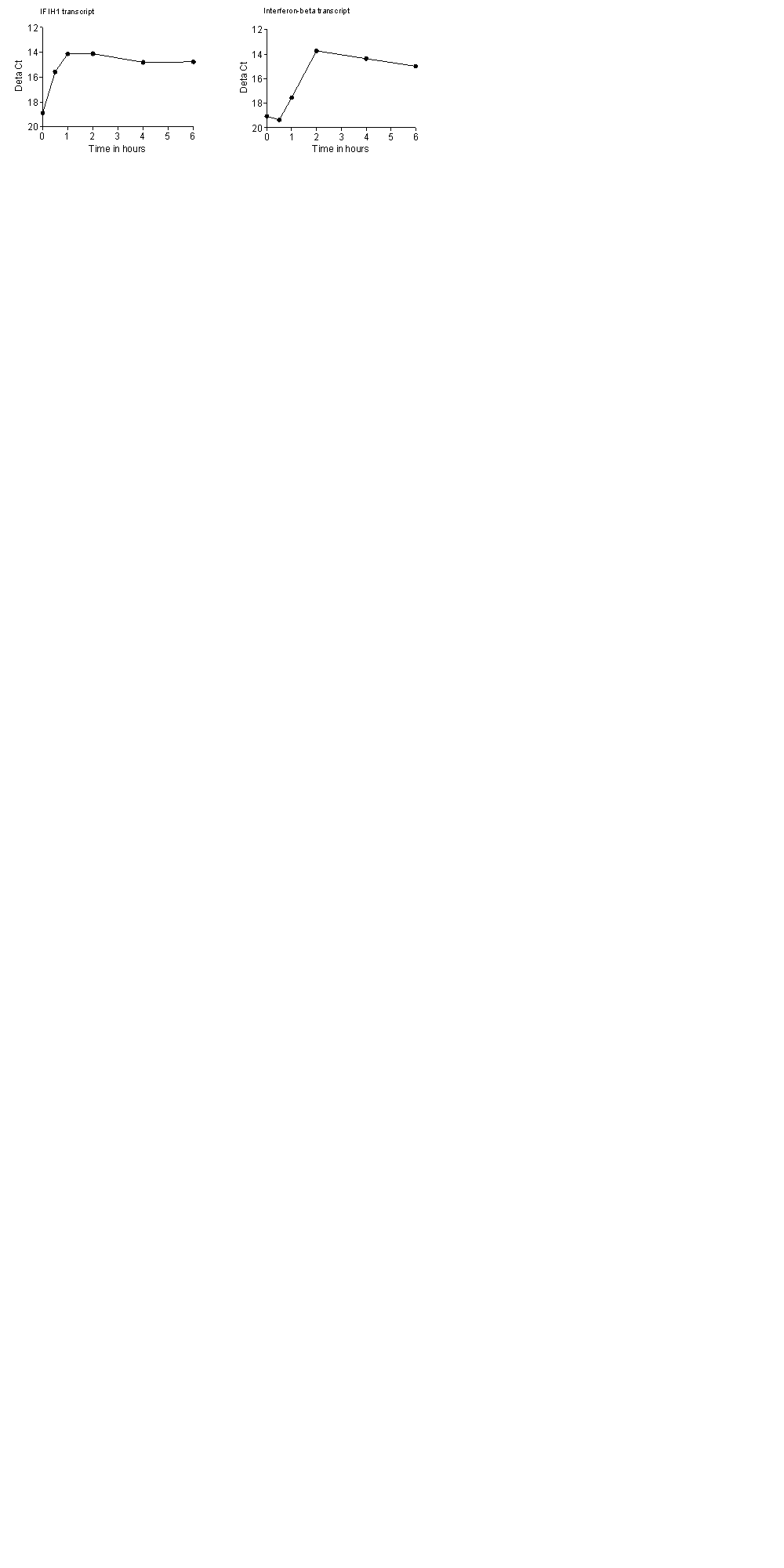

Supplement: Figure S4 — Pre mRNA levels of IFIH1 and IFNB1, the gene encoding IFN-β, increase and plateau in PBMCs stimulated with 1000 U/ml of IFN-β for 6 hours. Ct values for the gene specific assays are normalised using a qPCR assay for β2 microglobulin. (0.10 MB TIF) [file pone.0012646.s008.tif]

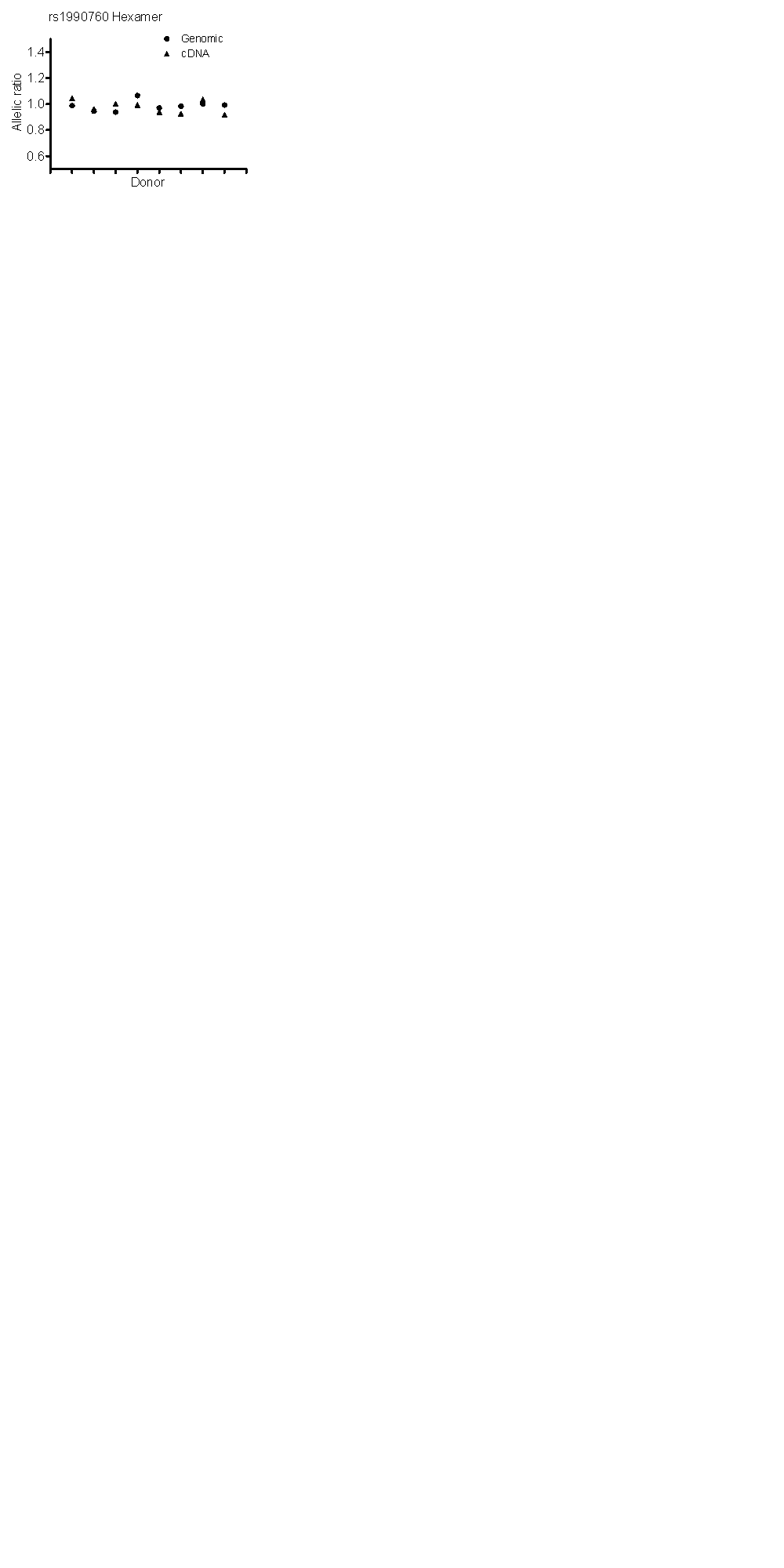

Supplement: Figure S5 — No allele specific bias is observed using the rs1990760 ASE assay in cDNA of fragmented RNA primed with hexamer from PBMCs stimulated with IFN-β (P = 0.64). (0.10 MB TIF) [file pone.0012646.s009.tif]

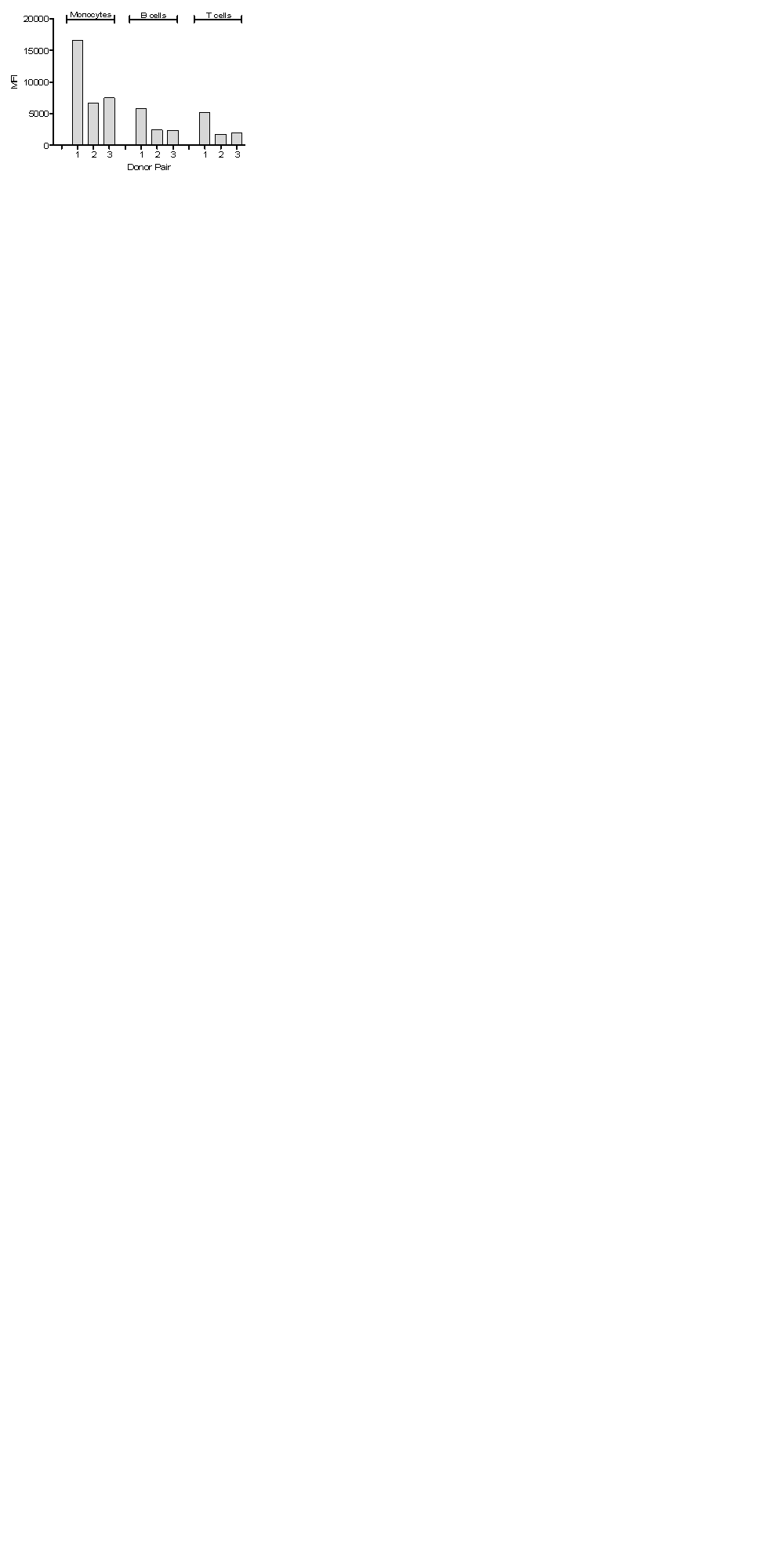

Supplement: Figure S6 — Mean fluorescence intensity (MFI) of IFIH1 protein expression in resting monocytes, B and T cells of the three paired control individuals. (0.10 MB TIF) [file pone.0012646.s010.tif]

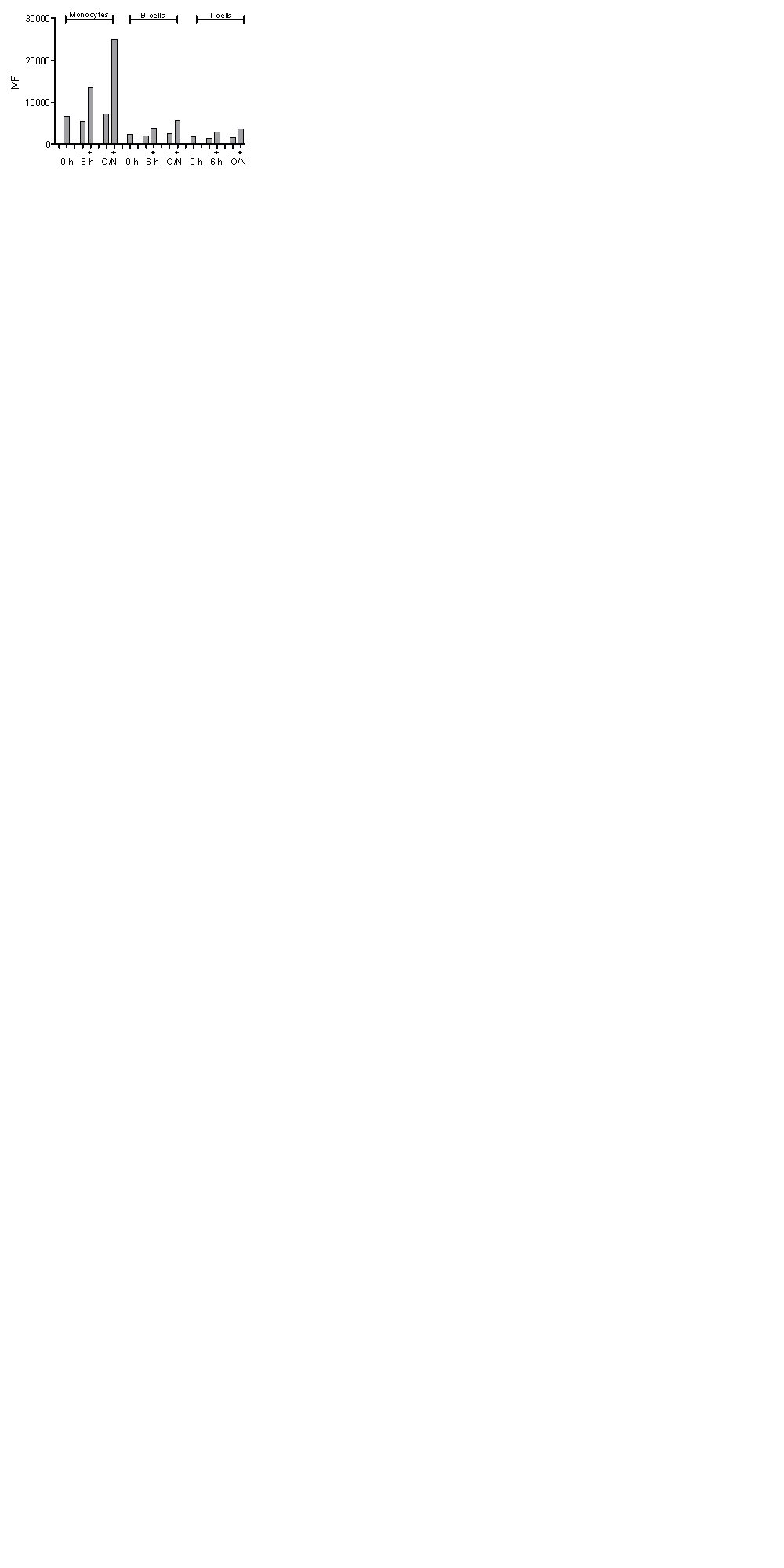

Supplement: Figure S7 — Expression of IFIH1 protein increases with IFN-β stimulation at 6 hours and overnight in monocytes, B and T cells of one paired control individual. (0.10 MB TIF) [file pone.0012646.s011.tif]

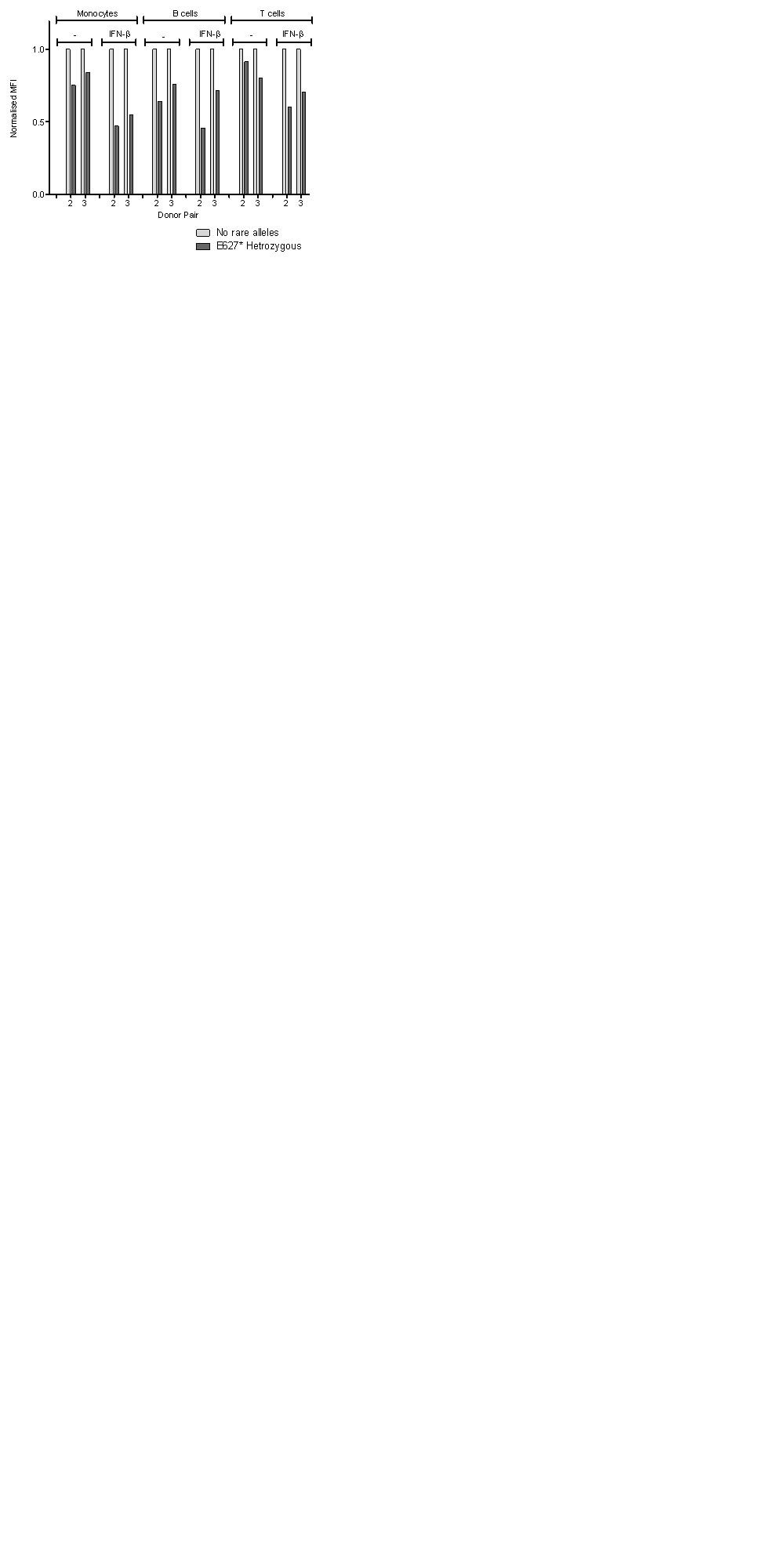

Supplement: Figure S8 — The reduced expression of IFIH1 protein due to the stop codon SNP, rs35744605 (Glu627X), is amplified with IFN-β stimulation within the cell subsets. Normalised MFI of IFIH1 expression within monocytes, B and T cells stimulated with 1,000 U/ml IFN-β overnight. The MFI of individuals heterozygous for the stop codon SNP is normalised to the MFI of the paired control individual. (0.11 MB TIF) [file pone.0012646.s012.tif]

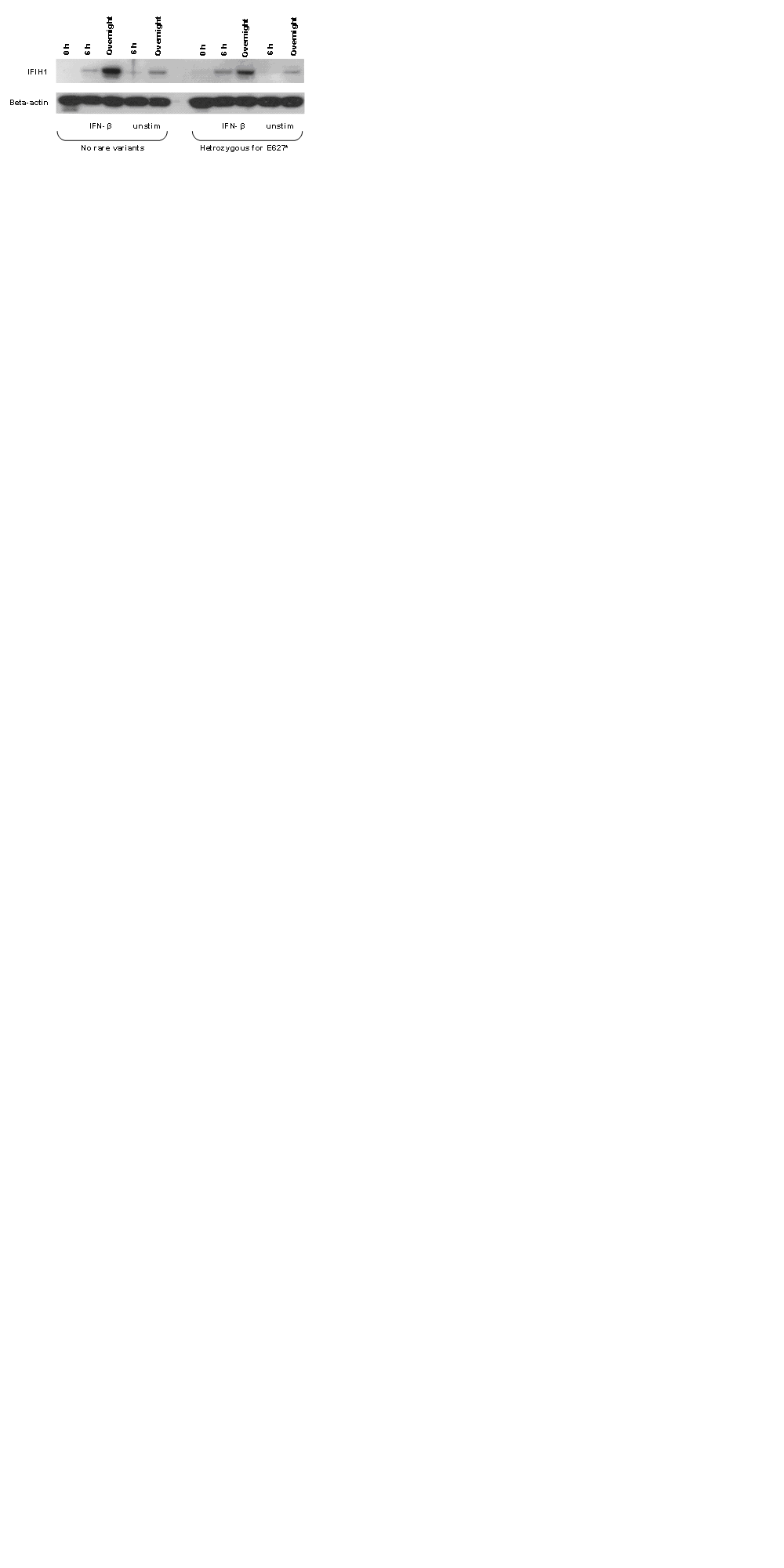

Supplement: Figure S9 — Reduced expression of IFIH1 due to the stop codon SNP, rs35744605 (Glu627X), may be observed by western blot of protein lysates from resting PBMCs and PBMCs stimulated with IFN-β for 6 hours and overnight. (0.10 MB TIF) [file pone.0012646.s013.tif]
